# The Medical Genome Reference Bank: a whole-genome data resource of 4000 healthy elderly individuals. Rationale and cohort design

**DOI:** 10.1038/s41431-018-0279-z

**Published:** 2018-10-24

**Authors:** Paul Lacaze, Mark Pinese, Warren Kaplan, Andrew Stone, Marie-Jo Brion, Robyn L. Woods, Martin McNamara, John J. McNeil, Marcel E. Dinger, David M. Thomas

**Affiliations:** 1 Department of Epidemiology and Preventive Medicine, School of Public Health and Preventive Medicine, Monash University, Melbourne, VIC Australia; 2 Garvan Institute of Medical Research, Sydney, NSW Australia; 3 St Vincent’s Clinical School, Faculty of Medicine, University of New South Wales, Sydney, NSW Australia; 4 Sax Institute, Sydney, NSW Australia

**Keywords:** Medical genetics, Clinical genetics, Population genetics

## Abstract

Allele frequency data from human reference populations is of increasing value for the filtering, interpretation, and assignment of pathogenicity to genetic variants. Aged and healthy populations are more likely to be selectively depleted of pathogenic alleles and therefore particularly suitable as a reference population for the major diseases of clinical and public health importance. However, reference studies of confirmed healthy elderly individuals have remained under-represented in human genetics. Here we describe the Medical Genome Reference Bank (MGRB), a large-scale comprehensive whole-genome data set of healthy elderly individuals. The MGRB provides an accessible data resource for health-related research and clinical genetics and a powerful platform for studying the genetics of healthy ageing. The MGRB is comprised of 4000 healthy, older individuals, mostly of European descent, recruited from two Australian community-based cohorts. Each participant lived ≥70 years with no reported history of cancer, cardiovascular disease, or dementia. DNA derived from blood samples has been subject to whole-genome sequencing. The MGRB has committed to a policy of data sharing, employing a hierarchical data management system to maintain participant privacy and confidentiality, while maximising research and clinical usage of the database. The MGRB represents a resource of international significance, which will be made broadly accessible to the clinical and genetic research community.

## Introduction

One of the key challenges in the interpretation of whole-genome sequencing (WGS) data for the diagnosis of inherited disease is discriminating rare candidate disease-causing variants from the large numbers of benign variants unique to each individual. Reference populations are powerful filters to distinguish pathogenic from population-based genetic variation, both clinically for Mendelian disorders [1, 2] and in research for studies of genetic disease [3].

The availability of population-based allele frequency data has been instrumental in enabling variant filtering, assignment of pathogenicity, and frequency-based estimates of penetrance in recent years [4–6]. Variant frequency data has facilitated the diagnosis and discovery of an unprecedented number of damaging variants affecting gene function, which can subsequently lead to disease. Population or allele frequency-based filtering has become a mainstay of clinical genetics. This was initially made possible by access to the International HapMap [6] and 1000 Genomes [5] data sets and then more recently by the Exome Aggregation Consortium (ExAC) and Genome Aggregation Database (GnomAD) [4]. All of these reference projects have been pivotal in influence on human genetics, primarily due to the common aspect of making variant frequency data readily available to users in the clinical and research communities. An increasing number of reference population sequencing projects are now underway worldwide, reflecting the need to understand the underlying genetic variation in different backgrounds, especially among non-European populations [7–11].

Despite the value provided by previous human genetic reference populations, each has been limited in some capacity. One of the most common and significant limitations has been the lack of detailed phenotypic or clinical information provided to the research community and rationale for sample ascertainment used. Such information is of particular importance for confirming or refuting the presence of genetic disease phenotypes in the reference population. Access to longitudinal clinical outcome data to interpret genetic variation considered to be pathogenic has also been lacking. For example, a cancer-free individual sampled at age 45 years may be included in a reference control population but then go on to develop cancer at a later age. This individual may carry a damaging cancer-predisposing germline variant; however, this individual may still be used as a negative control in many subsequent cancer studies.

When combined with the stochastic and environmentally dependent nature of disease phenotypes, identification of genetically risk-deplete controls is a critical aspect of defining high-confidence reference populations and subsequently achieving a better understanding of the genetic basis of common diseases. Only one whole-genome sequenced population comprised of individuals confirmed to be deplete of genetic disease phenotypes has been generated to date [12]. Depletion of disease phenotypes should decrease the burden of penetrant damaging variants that affect disease-related gene function. Such populations, such as the one we describe here, have increased power to act as negative controls for variant filtering and assignment of pathogenicity in studies focused on inherited or genetic disease.

Another challenge of reference populations is the relative size of the population. The larger a reference population is, the more likely the population will be to contain a particular rare variant. Therefore, larger sample sizes typically provide more robust population-based allele frequencies for rare variation. This is of critical importance, given most pathogenic alleles are rare, occurring at <1% in the population. Those of higher penetrance are often found at <0.1%. By volume, the majority of all single-nucleotide variants (SNVs) detected in the population are <1% frequency, as shown by recent population-based whole-exome sequencing and WGS studies, whereby singletons were by far the most abundant SNV frequency class [4, 11, 13].

One notable limitation of several human reference populations to date has been the aspect of data aggregation. Often genetic data have been provided by many different studies for aggregation efforts, with samples of varying ethnicity, age, and genetic background combined [4]. This is in contrast to a purpose-built reference cohort, confirmed to be depleted of disease from the outset, beyond a certain age. Data aggregation efforts have helped significantly to reach the higher sample numbers required for variant filtering based on disease population prevalence [14, 15]. However, data aggregation has typically not ensured a high quality or consistency of phenotypic information provided to the end user. By contrast, a purpose-built reference population of confirmed healthy elderly individuals, known to be deplete of genetic disease phenotypes, provides the end user with more confidence in the absence of genetic disease symptoms and clearer rationale for sample ascertainment. These are important features to consider in a reference population intended to be used as a negative control set.

Large genomic reference data sets containing both healthy and diseased individuals are valuable for population-based filtering based on allele frequency thresholds, corresponding to the prevalence of a related disease [15]. However, it is often difficult to determine exactly where to set this allele frequency cutoff, when prevalence and genetic architecture of diseases are unclear. This can be an issue especially for poorly characterised, rare, or phenotypically heterogeneous genetic conditions. Further, the logic of using population disease prevalence-based variant filtering presupposes knowledge about the genetics of the disease, including the genes involved, relative variant frequencies, and penetrance, which is not always known.

In the case of a confirmed disease-depleted cohort of the healthy elderly, there is the unique advantage of requiring no exact filter setting (or threshold) based on disease prevalence. This is because we can have confidence that the cohort does not contain any individuals affected by severe genetic disease, to an advanced age. Therefore, the frequency of fully penetrant causative variants for severe genetic disease can be reasonably assumed to be zero.

Achieving the unique combination of all features required for the optimal human genetic reference population is challenging but should include: large size by sample number, confirmation of health and age phenotypes (i.e. absence of disease) beyond an advanced age, whole-genome coverage, ability to detect complex and structural variation, availability of both genomic and phenotypic data, and measurement of genetic sequence variation using a consistent and compatible sequencing technology (see Table 1).

**Table 1:** Features of human genetic reference populations (according to public domain websites and peer-reviewed literature, February 2018)

	MGRB	ExAC [4]	GnomAD [4]	UKBB SNPs [28]	HLI - JCVI [13]	Wellderly STSI [12]	SweGen [11]	HGVD [7]
Approx. cohort size (Feb 2018)	4000	60,000	140,000	500,000	10,000	600	1000	3200
Purpose-built cohort (versus data aggregation)	✓	X	X	✓	✓	✓	✓	✓
Whole genome sequencing	✓	X	✓	X	✓	✓	✓	X
Ability to detect complex and SV	✓	X	✓	X	✓	X	✓	X
Phenotype data to confirm absence of disease	✓	X	X	✓	✓	✓	X	?
Confirmed healthy elderly population	✓	X	X	X	X	✓	X	X
Allele frequencies made readily accessible	✓	✓	✓	X	X	X	✓	✓
Formal data access and approval policy	✓	X	X	✓	X	X	✓	X
Access provided to individual VCFs	✓	X	X	X	X	X	X	X
*n* ≥ 4000 samples	✓	✓	✓	✓	✓	X	X	X
Consistent and compatible seq. technology	✓	✓	✓	X	✓	X	✓	✓

*MGRB* Medical Genome Reference Bank, *ExAC* Exome Aggregation Consortium, *GnomAD* Genome Aggregation Database, *UKBB SNPs* U.K. Biobank SNP data set, *HLI-JCVI* Human Longevity Inc - J. Craig Venter Institute, *STSI Wellderly* Scripps Translational Science Institute Wellderly study, *SweGen* Swedish Genome reference population project, *HGVD* Human Genetic Variation Database (Japan)

Here we present the rationale and cohort design of the first human reference population comprised of thousands of whole genomes from confirmed healthy elderly individuals depleted of common and rare genetic disease phenotypes. Samples for this project have been provided from two leading Australian community-based cohort studies, with access to phenotypic and clinical information, to confirm the absence of rare genetic disease, as well as depletion of common disease such as cardiovascular disease, dementia, and cancer, in all participants.

The Medical Genome Reference Bank (MGRB) has conducted WGS of 4000 healthy older adults. These individuals are participants of the ASPirin in Reducing Events in the Elderly (ASPREE) study, an international clinical trial for daily low-dose aspirin use in older people, coordinated by the Department of Epidemiology and Preventive Medicine at Monash University [16], or the 45 and Up study, the largest ongoing study of healthy ageing in the Southern Hemisphere, coordinated by the Sax Institute [17].

**Fig. 1:**
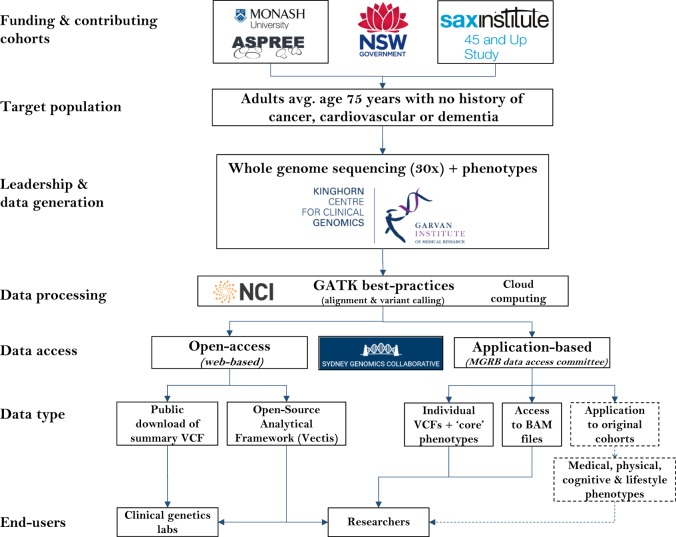
The Medical Genome Reference Bank: Project overview

Each MGRB sample has been sequenced using Illumina technology at a minimum 30× coverage. Data processing is conducted using WGS best practice pipelines (GATK-BWA). Resulting population allele frequency data is made openly accessible and downloadable via public website. Individual-level variant call files (VCFs), core phenotypes, and access to alignment files (BAMs) are open to application via the MGRB Data Access Committee. Access to additional clinical information and phenotype data is available via application to contributing cohorts [16, 17], via existing data access and governance arrangements. For MGRB schematic project overview, see Fig. 1.

## Methods/design

### Inclusion criteria

The MGRB is comprised of individuals consented through the biobank programs of two contributing studies, following protocols previously described [16–18]. Each sample is from an individual who has lived to ≥70 years with no reported history or current diagnosis of cardiovascular disease, dementia, or cancer, as confirmed by the participating studies at recent follow-up study visits. MGRB cohort demographics are shown in Table 2.

**Table 2:** MGRB summary demographics, by cohort

	45 and Up	ASPREE
Year of birth		
1910–1915	0	0
1915–1920	2	5
1920–1925	11	89
1925–1930	79	490
1930–1935	108	1388
1935–1940	181	1153
1940–1945	356	0
1945–1950	77	0
Sex		
Female	492 (60.4%)	1653 (52.9%)
Male	322 (39.6%)	1472 (47.1%)
Age at last follow-up (years; ~approx. 2016)		
70–75	324	0
75–80	235	349
80–85	132	1778
85–90	83	787
90–95	38	192
95–100	2	19

Beyond MGRB inclusion criteria, each sample from the ASPREE study is from a participant aged ≥75 years at the time of study enrolment, with no reported history of any cancer type. Each sample from the ASPREE study also meets the following criteria at the time of study enrolment; no clinical diagnosis of atrial fibrillation; no serious illness likely to cause death within the next 5 years (as assessed by general practitioner); no current or recurrent condition with a high risk of major bleeding; no anaemia (haemoglobin >12 g/dl males, >11 g/dl females); no current continuous use of other antiplatelet drug or anticoagulant; no systolic blood pressure ≥180 mm Hg and/or a diastolic blood pressure ≥105 mm Hg; no history of dementia or a Modified Mini-Mental State Examination (3MS) score ≤77 [19]; and no severe difficulty or an inability to perform any one of the six Katz activities of daily living [20].

Beyond MGRB inclusion criteria, each sample from the 45 and Up study also met the following criteria; no record of cancer diagnosis in the NSW Central Cancer Registry and no record of cancer diagnosis in the NSW Admitted Patient Data Collection.

### Phenotypic information

The following data are made available for all MGRB samples through open access: year of birth, gender, height, and weight. For samples from the ASPREE study, waist circumference, blood pressure, fasting blood glucose, and status of age-related macular degeneration are also made available through open access.

## Data generation

### Library preparation, DNA sequencing, alignment, and processing

WGS of MGRB samples has been performed using Illumina HiSeq X sequencers at the Kinghorn Centre for Clinical Genomics (KCCG) under clinically accredited conditions (ISO 15189). Paired-end Illumina TruSeq DNA Nano libraries were sequenced to one lane per sample. DNA sequences are mapped to Build 37 of the human reference genome and processed following the Genome Analysis Toolkit (GATK) best practices [21]. Indel realignment and base quality score recalibration of mapped reads are performed using GATK and best practices parameters; unmapped reads to be left unmodified. GATK HaplotypeCaller is used to generate g.vcfs from all single-lane realigned and recalibrated BAMs using recommended parameters. All of the raw data are processed through the Genome One Discovery pipeline (https://www.genome.one/discovery-genomics). Data are analysed using the Hail open-source framework for scalable genetic analysis (https://github.com/hail-is/hail).

### Phased data release plan

MGRB data will be generated, processed, and released in three phases (for timelines, see Table 3). Summary variant frequency data for the MGRB cohort is made available at the MGRB web portal: https://sgc.garvan.org.au. Complete genotype, phenotype, and raw data are available to qualified applicants following data access approval. Completion of each phase of sequencing will be followed by a public release of allele frequency data (Tier 1), including an update to the MGRB database, website, portal, and beacon.

**Table 3:** MGRB timeline for whole-genome sequencing and data release

MGRB	WGS target sample number	Progress timeline H1 = first half of year, H2 = second half of year		
		Sequencing completion	Tier 1 open data release	Tier 2/3 approval
Phase I	1500	H2 2016	H2 2016	H1 2017
Phase II	3000	H2 2017	H1 2018	H2 2018
Phase III	4000	H2 2018	H1 2019	H2 2019

### Data access

The MGRB Data Access Policy (DAP) summarises the governance applied to individual and institutional access (Table 4). Curated data will be openly accessible to the international research community through the MGRB website. Preliminary features will include a Beacon, as defined by the Global Alliance for Genomics and Health [22], extensive variant annotation, complex queries (including genetic annotations and genomic regions), visualisation of variant data (e.g. genome viewer/gene networks), and ultimately, analysis tools for assessing the genetic burden of individual variants and variant subsets.

**Table 4:** MGRB tiered Data Access Policy

Tier	1. Open Access	2. Controlled Access	3. Restricted Access
Access	Institutional email address required for MGRB data portal access (not required for Beacon) (www.sgc.garvan.org.au/mgrb)	Data Access Application (DAA) must be approved by the MGRB Data Access Committee (DAC)	DAA must be approved by the MGRB DAC and referred to the applicable cohort governing body for further approval
Clinical data	Basic demographic data are provided, genomic queries can be filtered according to these fields	Basic demographic data and minimal clinical information (where available) are provided per individual record	Comprehensive clinical data that is potentially specific to a participating cohort is provided per individual record
Genomic data	Beacon and pre-processed variant frequencies	Individual record data provided—either processed (VCF/gVCF format) or unprocessed (FASTQ or BAM format) (dependent on justification criteria being met)	

While basic demographic and phenotypic information will be incorporated into the MGRB data portal, researchers are invited to apply for access to comprehensive genotypic and clinical information to support high-level integrative analysis. To maintain participant privacy and confidentiality, while maximising MGRB utility, we have deployed a tiered data management system that determines the richness of data that is made available to researchers (as summarised in Table 3). This consists of Open access, Controlled access, and Restricted access tiers.

The restricted access tier (Tier 3) will involve access to more detailed phenotype and/or clinical information and requires application, project approval, and ethical approval from the ASPREE Presentations, Publications and Ancillary Studies Committee (PPA) or 45 and Up Data Access Committee. Notwithstanding internal priorities, and subject to collaborative agreement, both studies commit to fair and reasonable consideration of applications to provide access to restricted access tier data. Individual de-identified processed genomic data would be available to download from the MGRB after execution of a Data Transfer Agreement. In the case of the ASPREE study, any use of follow-up outcome data will need to take into account effects of randomisation to low-dose aspirin, which could impact health outcomes.

### Data transfer

Current MGRB policy is to provide processed VCFs (or gVCF) directly to approved applicants, via secure file transfer, subject to the MGRB DAP and Information Handling Statement (see www.sgc.garvan.org.au/mgrb). For access to raw data files (BAM or FASTQ), scientific rationale must be provided, after which approved access to BAM and/or FASTQ is provided via remote login to the MGRB server environment, hosted at the National Computation Infrastructure (NCI). This is to avoid transfer and duplication of extremely large file sizes.

### Open-source analytical framework (*Vectis*)

**Fig. 2:**
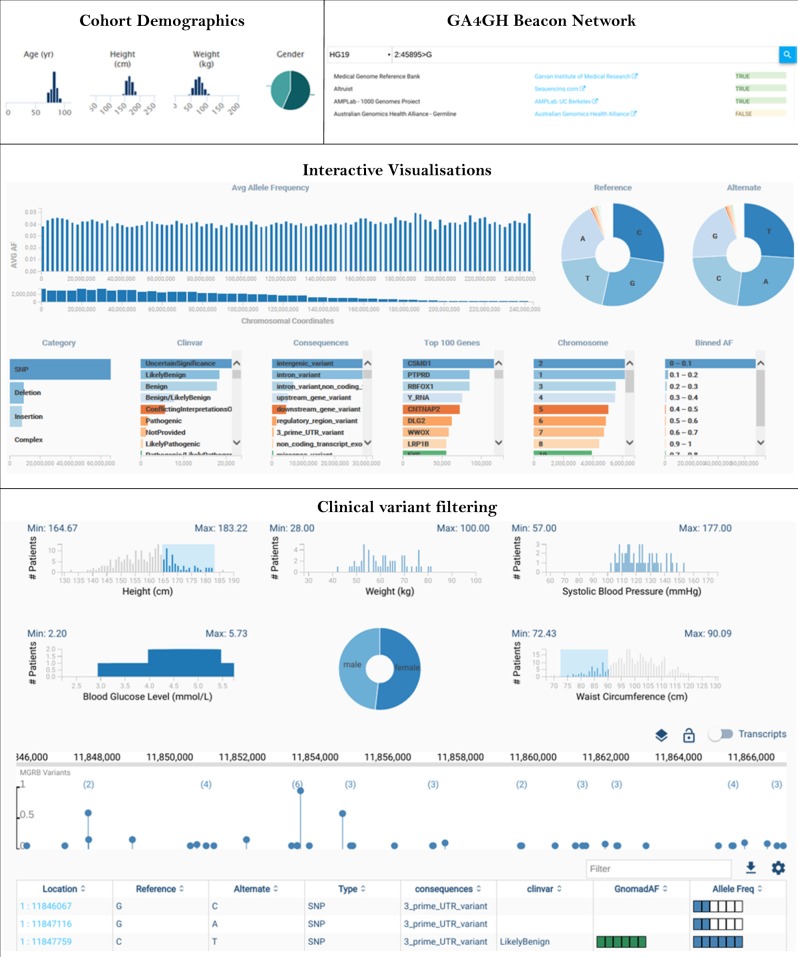
MGRB database functionality and Vectis platform

*Vectis* (a lever in Latin) is a custom-build software environment and collection of modules for the MGRB to support diverse users including clinicians, patients, and bench scientists as well as bioinformaticians for the analysis of patient cohorts of any size, comprising whole genomes, exomes, or gene panels. The *Vectis* modules are described in Fig. 2 and Table 5. *Vectis* is a collection of open source modules made available from GitHub (https://github.com/vectis-lab). The overall design comprises a Variant Store Abstraction Layer backed by MySQL. Elastic Gene Search is used for real time prompting of gene information, and the Ensembl REST API provides genomic reference information. The GA4GH Beacon Network is used for querying cohorts registered in the Beacon Network. Auth0 provides the identity and authentication service, with Sentry used to track unhandled errors. The *Vectis* Explore module backend relies on the in-memory GPGPU Database, which supports the cross-dimensional charts.

**Table 5:** Features of the *Vectis* open-source analytical framework

Feature	Description
Secure login	Two-factor authentication
Search	Querying of cohorts using chromosomal coordinates and gene annotations
Beacon	Integrated with the Global Alliance for Genomics and Health Beacon Network [19]
Explore function	Highly interactive, low latency exploration of cohorts. Explore currently supports the querying of 84 million variants in real time
Interactive graphics	Including lollipop plots of allelic frequencies as well as gene transcripts
Variant annotations	Including links out to the original supporting evidence
Scalable variant store	Enables authorised users to subset patients based on clinical attributes and query actual genotypes at the individual patient level

## Discussion

### Analysis aims

The overarching aim of the MGRB is to create a catalogue of genome-wide genetic variation in healthy, older individuals and make data readily available to the clinical genetics and research community. Secondary analysis aims of the MGRB project include, but are not limited to, detection of different variant classes, such as SNVs, insertions–deletions (Indels), structural variants, and copy number changes across the population; clustering of the cohort by ethnicity and other phenotypic factors; examining the frequency and type of clinically significant rare alleles, in relation to phenotypes; calculating polygenic risk scores for a range of conditions, and comparing these scores against population-based and disease-based cohorts; and measuring non-germline variation such as telomere length, mtDNA variation, and somatic changes in blood in relation to genomic ageing.

### Potential limitations and confounding factors

The MGRB study is limited by the size of the cohort (4000 individuals). Very rare genetic variants, many of which could be of clinical or biological interest, may therefore not be present in the data set, by chance. This limit of detection may restrict the sensitivity of MGRB for some applications, compared to larger data sets, such as ExAC and GnomAD [4]. However, for many applications, the cohort size of the MGRB is sufficient, with a very high probability of detection of variants with minor allele frequency (MAF) down to 0.1%. The benefits of the MGRB are balanced against the cohort’s size, and we believe that variant filtering based on a combination of MGRB and larger, less stringently ascertained cohorts will be the most robust approach to variant filtering.

The MGRB is an Australian cohort with a preponderance of Caucasian European ancestry. This limits its utility as a variant filter to matched disease populations. We also acknowledge it is still possible that some diseases with a partial genetic architecture may still manifest in the MGRB, such as some cancers with a late age of onset, beyond the time of sampling.

A biological limitation of the MGRB will be the variable penetrance of rare disease-predisposing variants, even in an elderly population [23, 24]. An important consideration is that, although the MGRB cohort is an aged, healthy group, it is still possible that rare clinically significant predisposing variants will be present, some of which will not be expressed through disease symptoms (non-penetrant). Most single gene predispositions, including familial cancers, are not fully penetrant, meaning that <100% of individuals with predisposing variants in such genes ever develop the associated clinical phenotype, even in older age [23]. The MGRB will give a unique opportunity to overcome the traditional ascertainment bias [25] of human genetics in this regard. However, the detection of a variant in the MGRB alone does not exclude its potentially pathogenic role, where variable penetrance could be due to genetic or environmental factors [26]. This is likely to be particularly important for assigning causality in common diseases, where polygenic effects are likely important. This caveat is something end-users of the data must keep in mind.

A data set of whole-genome sequences from 600 individuals aged >80 years has been published previously by the ‘Wellderly’ study [12]. Individuals in this study had no reported chronic diseases and were not taking chronic medications. There are important differences between this study and the MGRB. First, the number of samples in the MGRB will be significantly higher at 4000, adding much-needed power and sensitivity for detecting and filtering, rare variants. The MGRB will have an average limit of detection for rare variants at 1/8000 alleles (MAF = 0.000125) compared to 1/1200 alleles (MAF = 0.00002583). Second, significant resources within the MGRB have been allocated to ensuring data access, analytical frameworks, and data-sharing mechanisms for both WGS and phenotypic data. Third, there is the capacity to detect and report complex and structural genetic variation more readily. Fourth, the Wellderly study sequenced DNA using the Complete Genomics platform [27], not the technology used by most WGS or whole-exome sequencing of reference populations to date [4, 5, 7–9, 11, 13]. There are important technical considerations in the cross-compatibility of whole-exome and WGS data for generating population allele frequencies on different sequencing platforms or data processed using different bioinformatic pipelines [4].

### Implications

The MGRB has the potential to add another important resource to the clinical genetic and research community for filtering, annotation, and assignment of pathogenicity to genetic variants. The unique aspects of the MGRB will include: (1) focus on the healthy elderly, depleted of typical monogenic disease phenotypes; (2) age of the cohort, average >75 years, beyond the age of onset for most monogenetic conditions; (3) the availability and access to individual-level VCF and BAM data; and (4) the opportunity to access high-quality, comprehensive, longitudinal, clinical, and phenotypic information on sequenced samples [16, 17]. These factors will ensure that MGRB has a unique place alongside other reference populations in human genetics.

## Conclusion

The MGRB will be the first catalogue of whole-genome variation across thousands of healthy elderly individuals. This will provide an important data set, resource, and much-needed negative control population for clinical genetic and research use.
